# Chronic Macular Oedema as a Late MIRAgel-Related Complication

**DOI:** 10.3390/vision5040055

**Published:** 2021-11-08

**Authors:** Chung Shen Chean, Christina S. Lim, Hardeep-Singh Mudhar, Evangelos Lokovitis, Raghavan Sampath

**Affiliations:** 1 Department of Ophthalmology, University Hospitals of Leicester NHS Trust, Infirmary Square, Leicester LE1 5WW, UK; christina.sk.lim13@gmail.com (C.S.L.); elokovitis@hotmail.com (E.L.); raghavan.sampath@uhl-tr.nhs.uk (R.S.); 2 National Specialist Ophthalmic Pathology Service (NSOPS), Department of Histopathology, Royal Hallamshire Hospital, Sheffield S10 2JF, UK; hardeep.mudhar@nhs.net

**Keywords:** macular oedema, MIRAgel, scleral buckle, complication, vitreoretinal

## Abstract

Background: MIRAgel^®^ (MIRA, Waltham, MA, USA) is a hydrogel scleral buckle introduced in 1979 to treat rhegmatogenous retinal detachments. Its use was discontinued because late complications that require surgical removal were reported. Methods: Case report. Results: We report a case of left eye MIRAgel^®^ buckle surgery 28 years ago presenting with a tender palpable erythematous swelling at the lower lid, with marked conjunctival chemosis and progressive ophthalmoplegia. Imaging revealed a large, well-defined, horseshoe-shaped lesion in the extraconal space of the left orbit with globe distortion, with histological confirmation of an expanded hydrogel buckle. He recovered well following removal of the explant but developed chronic macular oedema a year later, which persisted despite sub-Tenon’s triamcinolone injections. Repeat imaging demonstrated remaining hydrogel explant. Macular oedema settled well upon successful surgical removal with no recurrence to date. Conclusion: Our case is the first to describe macular oedema as a late MIRAgel-related complication, with complete removal of the explant being the definitive treatment. Macular oedema indicates postoperative inflammation secondary to the remaining explant fragments. Given the friability of hydrolysed MIRAgel^®^, we recommend ophthalmologists to warn patients regarding the possibility of further inflammation in the globe or the orbit in case of incomplete removal.

## 1. Introduction

MIRAgel^®^ (MIRA, Waltham, MA, USA) is a hydrogel scleral buckle that was first introduced in 1979 as an alternative to silicone explants to treat rhegmatogenous retinal detachments [1]. It was initially thought that its soft, pliable characteristics could minimise scleral erosion, and it has the potential to absorb and release antibiotics, thereby preventing postoperative infections [2]. However, there were subsequent reports of the hydrogel undergoing hydrolytic degradation with progressive swelling of the explant causing severe complications, such as globe compression and intraocular invasion, strabismus, ptosis, scleral erosion, conjunctivitis, infection around the buckle as well as significant cosmetic problems [3,4,5,6,7,8,9,10,11,12]. These complications necessitated surgical removal of the buckle, which could cause concerns of retinal re-detachment and intraoperative scleral rupture [5]. Although its use was discontinued in the 1990s, its associated complications continue to be reported [2,13]. Due to the friable nature of the buckle material, complete surgical excision is known to be challenging.

Herein, we present a unique case of chronic macular oedema as a late MIRAgel-related complication. To our knowledge, this is the first case to be reported in the literature. 

## 2. Case Report

**Figure 1:**
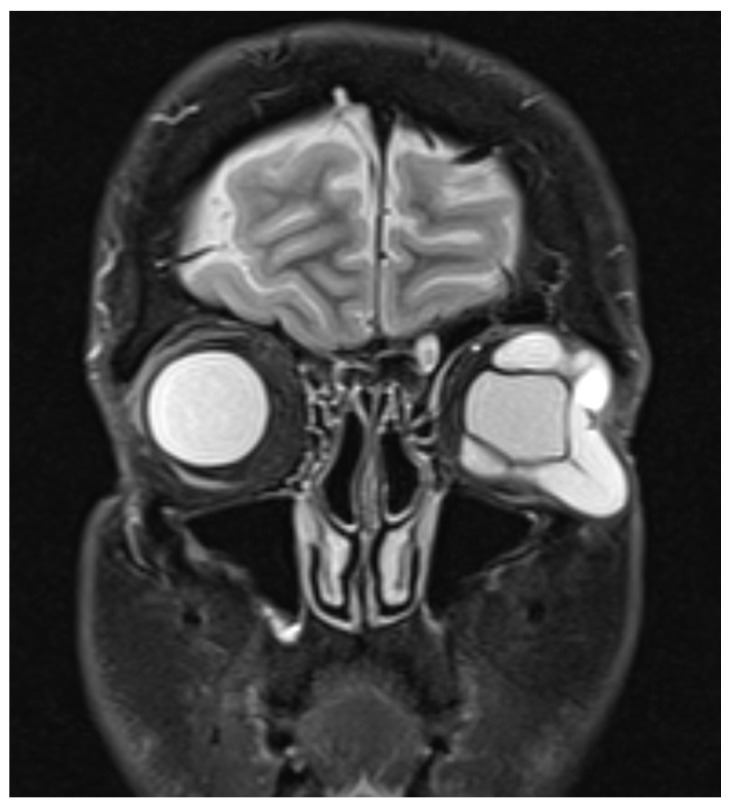
Coronal section of T2-weighted sequence of magnetic resonance imaging (MRI) of the orbits showing large tubular lesion in the extraconal space of the left orbit sparing the medial portion of the orbit with globe distortion.

**Figure 2:**
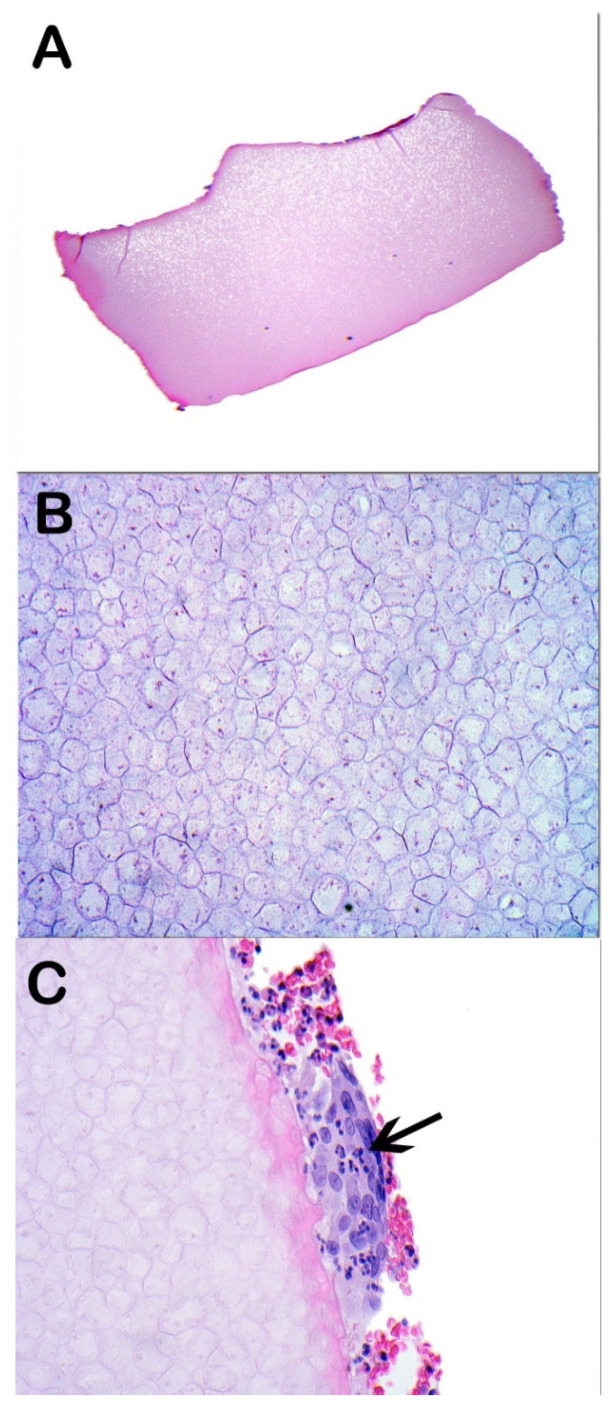
Histological microphotographs. (**A**) Low power magnification (×40) haematoxylin and eosin (H&E) stained section showing amorphous material. (**B**) Higher power magnification (×400) H&E showing bubbly basophilic material. (**C**) Higher power magnification (×400) H&E showing foreign body giant cells on the surface of the material (arrow).

A 62-year-old male presented with a 6-week history of gradual enlarging lesion in the left lower lid. This was accompanied by worsening pain and progressive ophthalmoplegia. He had no medical history to date, but he had a left eye (LE) retinal detachment 28 years ago, which was repaired using an encircling hydrogel scleral buckle. Visual acuity (VA) was 0.04 LogMAR in both eyes (BE) with normal pupillary examination and colour vision. A large, firm, ill-defined mass was palpable on the left lower lid with overlying erythema and marked conjunctival chemosis. There was a complete restriction in all positions of gaze in the LE. Exophthalmometry measurements revealed 2 mm of LE proptosis, and the left orbit was firm on retropulsion. Intraocular pressure (IOP) was 14 mmHg in BE. Posterior segment examination revealed healthy optic nerves and retina with no choroidal folds. There was no evidence of cervical lymphadenopathy. Urgent magnetic resonance imaging (MRI) of the orbits revealed a large, well-defined, tubular horseshoe-shaped lesion in the extraconal space of the left orbit, sparing the medial portion of the orbit with globe distortion which did not enhance on post-contrast sequences (Figure 1). Urgent incisional biopsy revealed multiple fragments of opaque grey-blue gelatinous material, and histology confirmed acellular material of an expanded hydrogel retinal detachment encircling band (Figure 2). 

Following the histology report, complete surgical excision of the hydrogel implant was attempted. A subconjunctival incision on the left lower lid and further blunt dissection were carried out to expose the thick white capsule that covered the gelatinous material of the expanded hydrogel. The gelatinous friable hydrogel buckle was removed with gentle use of suction, taking care not to cause scleral rupture or perforation. Multiple areas of scleral thinning were noted after the removal of the hydrogel buckle. The same technique was used for the lateral and superior quadrants to ensure complete removal of the explant. The conjunctiva was closed with a 7-0 Vicryl suture. Additional material sent for histopathological analysis confirmed the previous result. There was no intraoperative complication, but transient hypotony occurred postoperatively. At one week postoperative follow-up, there was a complete resolution of symptoms with full extraocular motility. VA was 0.04 LogMAR for BE. IOP was 15 mmHg in the right eye (RE) and 10 mmHg in the LE, with no signs of inflammation or infection. The patient recovered well and was subsequently discharged as he remained asymptomatic after being followed up for 4 months.

**Figure 3:**
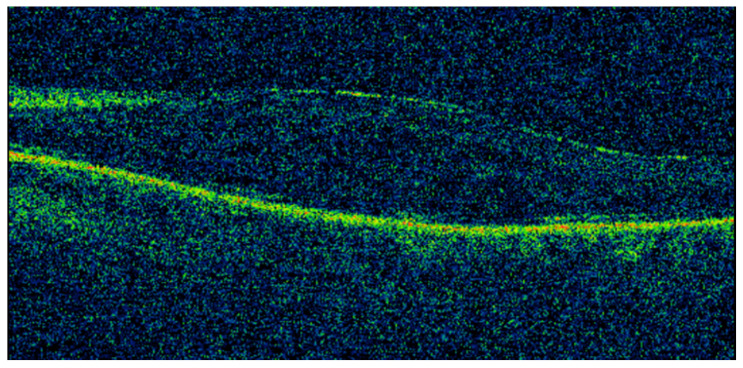
Optical coherence tomography (OCT) of the macula of the left eye with poor signal due to cataract but showing macular oedema.

**Figure 4:**
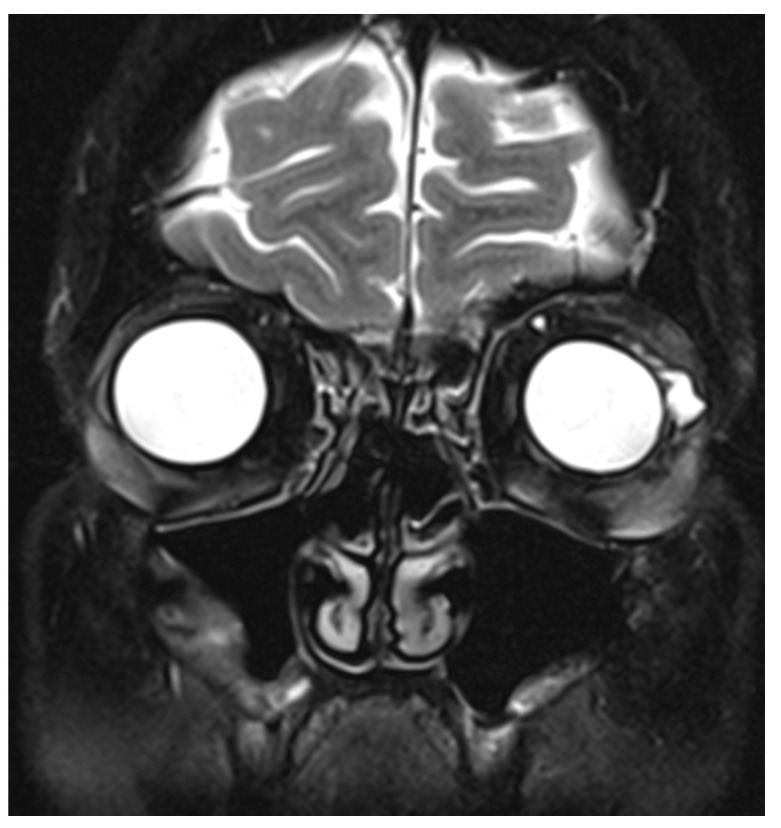
Coronal section of T2-weighted sequence of magnetic resonance imaging (MRI) of the orbits showing a cystic elevation above the left lateral rectus, likely to be a remnant of exposed hydrogel explant.

A year later, the patient was referred back with reduced vision in the LE. VA was 0.02 LogMAR on the RE and 0.96 on the LE. There was posterior subcapsular cataract and macular oedema in the LE, as shown in Figure 3. Despite receiving two sub-Tenon triamcinolone injections, macular oedema persisted. Considering the patient’s complex past ophthalmic history, a repeat MRI imaging of the orbits was organised, which revealed a cystic elevation above the left lateral rectus, likely to be a remnant of exposed hydrogel explant (Figure 4). The patient underwent further surgical excision as the remaining hydrogel explant was thought to cause the macular oedema. Following further surgical removal of the hydrogel, the macular oedema settled well with no recurrence to date. The patient was listed for cataract surgery of the LE, with the expectation of a good visual prognosis.

## 3. Discussion

Hydrogel scleral buckles (MIRAgel^®^) were popularised in the 1980s as they were perceived as an improvement upon the prior available silicone buckles [14]. As time passed, its complications became apparent, some of which had devastating ophthalmic consequences such as intraocular erosion and invasion, extrusion of the buckle, as well as endophthalmitis [5,15]. The expansion of the hydrogel implant was linked to hydrolytic degradation of multiple esters in the material, causing swelling and a change in its consistency to a more friable, gel-like object [16]. The friable nature makes them difficult to be removed surgically and is fraught with complications, as it often breaks into many small pieces when removal is attempted. The entire buckle often cannot be removed, and residual fragments are commonly left behind. These residual pieces have been shown to lead to significant inflammation and other problems [17]. 

To our knowledge, we believe this to be the first report of a hydrogel (MIRAgel^®^) explant causing macular oedema, which is shown to resolve upon removal of the hydrogel buckle. It is not uncommon for MIRAgel to cause an inflammatory reaction. Previous reports demonstrated scleral erosions, external inflammation that could mimic cellulitis, and granulomatous inflammation presenting as an orbital tumour or orbital cyst [17]. These findings were reflected in our patient, who initially presented with superficial eyelid redness, swelling, and conjunctival chemosis, with multiple areas of scleral thinning found intraoperatively. There was also a relevant case report of MIRAgel-related complication presenting as uveitis [13]. 

We believe the macular oedema in the current case we reported was inflammatory in aetiology, and was related to the residual exposed hydrogel buckle, as proven by imaging and histological analysis. Our patient initially presented with good VA in the affected eye and had no other relevant past ophthalmic or medical history that might have explained the macular oedema. Besides, the macular oedema, in this case, was also resistant to sub-Tenon corticosteroid treatment. It appears that the initial attempt to surgically remove the hydrogel caused some fragments to migrate to a more posterior part of the orbit, hence causing macular oedema. Given the well-reported inflammatory property of MIRAgel and the difficulty in its removal, we recommend ophthalmologists warn the patients regarding the possibility of further inflammation in the globe and the orbit in case of incomplete removal. 

Various surgical techniques have been proposed to allow complete removal of the hydrolysed explant to prevent further complications. We used a suction device to assist in pushing manoeuvres, similar to the technique proposed by Richards and Meyer [13]. Although they managed to remove the scleral buckle successfully in four patients using this technique, we found this challenging, and the patient in our case suffered late complications related to the remaining explant fragments. Several other techniques were proposed in the literature, including the pulling on the implant with a cryoprobe [18], floating the implant out of the capsule with balanced salt solution [19], consolidating the implant with boric acid to facilitate removal in one piece [20], or most recently, the modified suction-assisted removal technique [21]. It is uncertain whether which technique works best. Still, ophthalmologists should be mindful to examine for any evidence of postoperative inflammation, which, if present, should raise suspicion of remaining explant fragments. 

In conclusion, our case is the first to describe macular oedema as one of the complications related to the hydrogel scleral buckle. This may be due to postoperative inflammation secondary to the remaining explant fragments. We have shown that it can resolve upon complete removal of the explant. Further studies are needed to investigate any surgical technique that can remove the explant safely and successfully so that it does not cause any further long-term complications. 

## Data Availability

Not applicable.
